# Under-recognized Hypoparathyroidism in Thalassemia

**DOI:** 10.4274/jcrpe.0020

**Published:** 2018-11-29

**Authors:** Hataitip Tangngam, Pat Mahachoklertwattana, Preamrudee Poomthavorn, Ampaiwan Chuansumrit, Nongnuch Sirachainan, La-or Chailurkit, Patcharin Khlairit

**Affiliations:** 1 Mahidol University Faculty of Medicine, Ramathibodi Hospital, Department of Pediatrics, Bangkok, Thailand; 2 Mahidol University Faculty of Medicine, Ramathibodi Hospital, Department of Medicine, Bangkok, Thailand

**Keywords:** Thalassemia, hypoparathyroidism, hypocalcemia, iron overload, fibroblast growth factor-23

## Abstract

**Objective:**

Symptomatic hypoparathyroidism [symptomatic hypocalcemia without elevated serum parathyroid hormone (PTH)] in patients with thalassemia is relatively rare. Asymptomatic mild hypocalcemia without elevated PTH, which is considered hypoparathyroidism, may be more common but under-recognized.

**Methods:**

Sixty-six transfusion-dependent thalassemic patients and 28 healthy controls were enrolled. Serum calcium (Ca), phosphate (P), creatinine (Cr), albumin, intact PTH, 25-hydroxyvitamin D (25-OHD), plasma intact fibroblast growth factor-23 (FGF-23), urinary Ca, P and Cr were measured. Tubular reabsorption of P was calculated.

**Results:**

Thalassemic patients had significantly lower median serum Ca levels than the controls [8.7 (7.8-9.7) vs 9.6 (8.7-10.1) mg/dL, p<0.001]. Hypoparathyroidism was found in 25 of 66 (38%) patients. Symptomatic hypoparathyroidism was not encountered. Thalassemic patients also had significantly lower median plasma FGF-23 levels than the controls [35.7 (2.1-242.8) vs 53.2 (13.3-218.6) pg/mL, p=0.01]. In patients with hypoparathyroidism, median plasma FGF-23 level was significantly lower than that of normoparathyroid patients [34.8 (2.1-120.0) vs 43.1 (3.2-242.8) pg/mL, p=0.048]. However, serum P, Cr, intact PTH and 25-OHD levels were not significantly different in the two groups.

**Conclusion:**

Hypoparathyroidism was not uncommon in patients with transfusion-dependent thalassemia treated with suboptimal iron chelation. Plasma intact FGF-23 level in hypoparathyroid patients was lower than that of normoparathyroid patients.

**What is already known on this topic?**

Symptomatic hypoparathyroidism in patients with transfusion-dependent thalassemia is relatively rare. Data on prevalence of asymptomatic hypoparathyroidism are also scanty.

**What this study adds?**

Hypoparathyroidism in patients with thalassemia is not uncommon. In comparison with patients with normoparathyroidism, plasma fibroblast growth factor 23 was lower in patients with hypoparathyroidism. Screening for asymptomatic mild hypocalcemia without elevation of parathyroid hormone should be considered in transfusion-dependent thalassemia for early detection and proper treatment.

## Introduction

Thalassemia is an inherited disease caused by abnormal hemoglobins. It leads to ineffective erythropoiesis and increased peripheral hemolysis. Regular blood transfusion is inevitable in patients with moderate to severe thalassemia. Overt hypoparathyroidism in thalassemia is relatively rare (1). However, asymptomatic hypoparathyroidism has been rarely reported, although its incidence was as high as 42% in one study (2). Previous studies have shown that hypoparathyroidism was primarily associated with iron overload (3,4,5,6).

Relatively high serum phosphate (P) levels in children and adults with thalassemia were reported in several studies (7,8,9,10). Our previous study also demonstrated that serum P levels in transfusion-dependent thalassemia had a trend to be higher than those in non-transfusion dependent thalassemia cases, but not significantly so (p=0.081) (11). In transfusion-dependent thalassemia, high P loading due to regular blood transfusions, hemolysis and hypoparathyroidism contribute to elevated serum P levels (12).

Fibroblast growth factor-23 (FGF-23), a phosphaturic hormone, is mainly synthesized and secreted by osteoblasts and osteocytes in response to hyperphosphatemia and elevated 1.25-dihydroxyvitamin D (1.25-(OH)2D) concentration (13). FGF-23 acts at the renal tubular cell level to reduce P reabsorption. In addition, FGF-23 inhibits 1a-hydroxylase, leading to a reduction in formation of 1.25-(OH)2D (14). FGF-23 also reduces parathyroid hormone (PTH) secretion from the parathyroid glands, thereby attenuating the PTH-mediated phosphaturic effect (15). However, the mode of FGF-23- P axis control in thalassemia has not been elucidated.

Our previous histomorphometric study demonstrated that iron deposits in thalassemic bones impaired bone mineralization and reduced bone formation (16). *In vitro *studies demonstrated that excessive iron inhibited osteoblast proliferation and differentiation (17,18). Therefore, iron accumulation in thalassemic bones may compromise FGF-23 production by osteoblasts and osteocytes.

We therefore hypothesized that asymptomatic hypoparathyroidism might be common but under-recognized in patients with thalassemia. In addition, impaired FGF-23 production, secondary to iron deposits in bones, might partly contribute to elevated serum P in thalassemia. Our study aimed to determine serum calcium (Ca), P and 25-hydroxyvitamin D (25-OHD), PTH and plasma FGF-23 levels in transfusion-dependent thalassemic patients.

## Methods

Children and adolescents with transfusion-dependent thalassemia attending the Hematology Clinic at the Department of Pediatrics, Faculty of Medicine, Ramathibodi Hospital, Mahidol University, Bangkok, Thailand were enrolled in the study. Most of these transfusion-dependent thalassemic patients had received a standard regular blood transfusion therapy every 3-4 weeks to maintain their hemoglobin levels at 9-10.5 g/dL. Desferrioxamine was the only iron chelating agent used in patients who had a serum ferritin level greater than 1000 ng/mL. Additional oral iron chelators such as deferiprone and deferasirox have been used in the past five years. All patients received daily folic acid and multivitamin supplementation. Each tablet of multivitamin contains 400 IU of vitamin D2. Patients with known underlying conditions including hypoparathyroidism, renal disease and acute hemolysis, and patients who had been taking other medications affecting Ca, P and vitamin D metabolism, were excluded. The controls were healthy children who attended the day camp regularly arranged by our hospital during the end of each school semester. All these controls were the offspring of hospital personnel. None of them had been taking medications known to affect Ca, P and vitamin D metabolism.

Anthropometric measurements were performed at the time of enrollment. Measurements included weight to the nearest 0.1 kg measured using a digital weighing scales; height to the nearest 1 mm, measured using a Harpenden stadiometer (Holtain Ltd, Crymych, Dyfed, Wales). Z-scores of height and weight were calculated using the National Standard Growth Curve of the Ministry of Public Health, Thailand. The Z-score of body mass index (BMI) was calculated using the World Health Organization BMI for age and sex. Median serum ferritin was determined using serum ferritin levels periodically obtained during routine clinic visits. Cumulative iron loading was estimated from cumulative volume of packed red cell (PRC) transfusion as follows:

Cumulative iron loading (mg)=volume (mL) of PRC given x hematocrit of PRC x 1.16 (19).

Fasting blood samples were obtained in thalassemic patients and controls for determination of serum Ca, P, Cr, albumin, intact PTH, 25-OHD and plasma intact FGF-23 levels. In thalassemic patients, fasting blood samples were obtained on the day of transfusion just before the scheduled transfusion. Simultaneous spot morning urine samples for Ca, P and Cr in thalassemic patients were obtained. Serum PTH and 25-OHD were measured by chemiluminescence assay (Roche Diagnostics GmbH, Mannheim, Germany). Corrected serum Ca, tubular reabsorption of P (TRP) and ratio of tubular P (TP) reabsorption to the glomerular filtration rate (TP/GFR) were calculated using the following formulas:

Corrected serum Ca (mg/dL) = Total serum Ca (mg/dL) + 0.8 x [4 – serum albumin (g/dL)]

TRP (%) = {1- [(urine P / serum P) / (urine Cr / serum Cr)]} x 100

TP/GFR (mg/dL) = Serum P – (urine P x serum Cr / urine Cr) (20)

Definitions used in this study: Hypocalcemia = corrected serum Ca <8.5 mg/dL; normocalcemia = corrected serum Ca 8.5-10.4 mg/dL; hypoparathyroidism = hypocalcemia without elevated intact PTH (reference range: 20-75 pg/mL).

Plasma intact FGF-23 was measured in duplicate by a commercial the enzyme-linked immunosorbent assay (ELISA) kit (2nd generation human intact FGF-23 ELISA kit, (Immutopics Inc, San Clemente, CA). The intra-assay coefficient of variation (CV) was 2.0-4.1% and the inter-assay CV was 3.5-9.1%. The lower limit of detection was 1.5 pg/mL.

The study was approved by the Ethics Committee of the Faculty of Medicine, Ramathibodi Hospital, Mahidol University (ethics approval no. MURA2013/24 Np1). Informed consent was obtained from the patients and their legal guardians.

### Statistical Analysis

Statistical analyses were performed using the software package SPSS 15.0 (SPSS, IBM Inc., Chicago, USA). All parameters were not normally distributed determined by Kolmogorov-Smirnov test and therefore, they were presented as median (range). Comparison between the patient and control groups was performed using Mann-Whitney U test. Pearson’s correlation was used to determine the correlation between two variables. A p value of less than 0.05 was considered statistically significant.

## Results

Sixty-six transfusion-dependent thalassemic patients, with a median (range) age of 13.5 (5.1-23.2) years, and 28 healthy controls, median (range) age of 8.9 (4.8-17.2) years participated in the study. Sixty of these patients were β-thalassemia/hemoglobin E (β-thal/E) cases and six were patients with β-thalassemia homozygote (β-major) disease. Twelve patients were splenectomized. All patients had been receiving monthly PRC transfusions and iron chelation therapy, including desferrioxamine in combination with either deferiprone or deferasirox. In comparison with the controls, thalassemic patients were significantly older, but their Z-scores for weight, height and BMI were lower. Thalassemic patients had significantly lower corrected serum Ca and plasma intact FGF-23 levels than those of the controls. No significant differences in serum P, PTH and 25-OHD levels were found between the two groups (Table 1). There were no significant differences in corrected serum Ca, P, 25-OHD, PTH and plasma intact FGF-23 levels between patients with β-thal/E and β-major. Hypoparathyroidism (corrected serum Ca 7.5-8.4 mg/dL and no elevation of PTH) was found in 25 of 66 (38%) thalassemic patients. None of the controls had hypocalcemia. Most thalassemic patients (94%) had normal vitamin D status (25-OHD ≥20 ng/mL). Only 4 of 66 (6%) patients (3 β-thal/E, 1 β-major) had mild vitamin D insufficiency (25-OHD, range 18.6-19.7 ng/mL). All but one of the controls (27 of 28) had normal vitamin D status (one child had mild vitamin D insufficiency, 25-OHD 16.8 ng/mL).

Twenty-five thalassemic patients had asymptomatic mild hypocalcemia. All these patients who had either an inappropriately low or normal serum PTH were considered to have hypoparathyroidism. The remaining 41 patients had normal serum Ca and PTH levels. There were no significant differences in gender, age, Z-scores of weight, height and BMI and ages of onset of transfusion and chelation therapy between patients with hypoparathyroidism and those with normoparathyroidism. In the hypoparathyroid group, the lowest level of 25-OHD was 18.6 ng/mL; almost all patients had vitamin D sufficiency (median 27.6 ng/mL). In comparison with normoparathyroid patients, median plasma intact FGF-23 was slightly but significantly lower in the hypoparathyroid group (43.1 vs 34.8 pg/mL, p=0.048). There were no significant differences in the serum P levels of these two groups. Cumulative iron loading was greater, while serum ferritin was lower in the hypoparathyroid group (Table 2). Serum Ca level had a negative correlation with duration of transfusions in hypoparathyroid patients (r=-0.45, p=0.022), but had no correlation in the normoparathyroid group.

## Discussion

The present study demonstrates asymptomatic hypoparathyroidism in transfusion dependent thalassemia. Previous studies reported low prevalences of hypoparathyroidism, ranging from 0.5 to 7.6% (5,12,21). Those prevalences primarily represented overt or symptomatic hypoparathyroidism. This cross-sectional study looked at Ca-P metabolism in patients with transfusion-dependent thalassemia. No patients had symptoms of hypocalcemia or a history of fractures. The prevalence of hypoparathyroidism was 38% in this study, which suggests a high prevalence of unrecognized, asymptomatic hypoparathyroidism. This is in line with previous studies of Mostafavi et al (22) and Adil et al (23) that reported hypoparathyroidism in 22.7% and 35.3% of thalassemic patients, respectively. Previous studies demonstrated that hypoparathyroidism was associated with high serum ferritin levels (3,4,5,6). A serum ferritin level higher than 2,500-3,000 ng/mL has been demonstrated to be associated with higher frequency of hypoparathyroidism (5,6). In addition, Belhoul et al (6) also reported that patients with a serum ferritin >2,500-3,000 ng/mL were 3.27 times more likely to develop hypoparathyroidism. However, no relationship between hypoparathyroidism and serum ferritin has been reported (24,25). In the present study, the patients had modestly elevated, median serum ferritin level of 1333 ng/mL and the median serum ferritin level in patients with hypoparathyroidism was lower than that of normoparathyroid patients. This finding can be explained by recent additional iron chelation treatment with oral deferiprone and deferasirox. Iron chelation improved strikingly with this treatment, resulting in rapid reduction of serum ferritin. However, tissue iron accumulation may still persist to a degree. In fact, serum ferritin levels were greater than 3,000 ng/mL in our transfusion-dependent thalassemics during the past 5-10 years when only desferrioxamine injection had been used (11).

Previous studies also demonstrated that serum ferritin may not be a reliable indicator of tissue iron overload (24,26). In patients with suboptimal iron chelation therapy, the amount of iron from PRC transfusion may better reflect tissue iron accumulation (27). Hence, our patients with hypoparathyroidism had higher cumulative iron loading than the normoparathyroid patients, despite lower serum ferritin levels. The cause of hypoparathyroidism is likely due to iron deposition in parathyroid glands, as previously reported (1,24,28,29).

FGF-23 is a phosphaturic hormone secreted by osteoblasts and osteocytes in response to elevated serum P. Elevated serum FGF-23 levels have been demonstrated in hypoparathyroid patients secondary to other causes such as parathyroidectomy, thyroidectomy or accidental parathyroidectomy and transient hypoparathyroidism in the offspring of hyperparathyroid mothers (30,31,32). In contrast, our hypoparathyroid thalassemic patients did not have elevated FGF-23 levels. A previous study reported that excessive iron disturbed the metabolism of mouse osteoblastic cells (17). In addition, ferric iron was shown to inhibit osteoblast proliferation, differentiation and mineralization. Moreover, the inhibition of human osteoblast activity was concentration-dependent (18). Iron overload inevitably occurs in transfusion-dependent thalassemic patients and iron accumulation in thalassemic bones has also been demonstrated (16,33). Thus, FGF-23 production by osteoblasts and osteocytes could be compromised in thalassemic patients. Our study showed that plasma intact FGF-23 level was significantly higher in the controls as compared with the thalassemic group. This finding could reflect an impaired FGF-23 production among thalassemic patients. Median plasma FGF-23 level in patients with hypoparathyroidism was significantly lower than that of normoparathyroid patients (34.8 vs 43.1 pg/mL, p=0.048) although serum P levels and serum PTH levels were comparable. These findings suggest that FGF-23 response in patients with hypoparathyroidism might be impaired. One would expect to see elevated serum P levels in these hypoparathyroid patients because of impairment of both phosphaturic hormones, PTH and FGF-23. The reason for an absence of elevated serum P in these patients is unclear and merits further investigation.

Iron deficiency, an opposite condition to iron overload state, has been reported to be associated with elevated serum FGF-23 levels in patients with autosomal dominant hypophosphatemic rickets (ADHR), in the elderly population and in undernourished Gambian children (34,35,36). In addition, iron deficiency upregulated *Fgf23* mRNA in bones of *Fgf23* knock-in mice and consequently led to an ADHR phenotype (37). Improvement of iron status following iron supplementation was associated with a decrease in serum FGF-23 level in undernourished Gambian children and patients with ADHR (36,38). Moreover, the latter had a complete loss of biochemical ADHR phenotype following iron supplementation (38). The mechanism of iron status in influencing FGF-23 concentration remains to be elucidated. However, to our knowledge, the impact of an iron overload state on FGF-23 level, secondary to thalassemia or hereditary hemochromatosis, has not been reported. One might speculate that an iron overload leads to a decrease in FGF-23 production in an opposite direction to the effect of iron deficiency. Our previous study demonstrated “iron-associated focal osteomalacia” in bone histology of patients with thalassemia (16). Osteoblasts and osteocytes could be disturbed by iron accumulation in bones and thus lead to impaired FGF-23 production. Further studies are required to assess the effects of iron overload on the synthesis, secretion and metabolism of FGF-23.

### Study Limitations

There were some limitations of this study. First, the sample size was relatively small. Second, the control and thalassemic groups were not matched for age and pubertal maturation level. However, previous studies reported no age- and puberty-associated changes in FGF-23 levels (39,40). Third, serum 1.25-(OH)_2_ vitamin D was not measured. Thus, pathophysiological changes of serum 1.25-(OH)_2_ vitamin D related to plasma FGF-23 during hypoparathyroidism or normoparathyroidism could not be assessed.

## Conclusion

Hypoparathyroidism was not uncommon in patients with transfusion-dependent thalassemia treated with suboptimal iron chelation. Plasma FGF-23 level in patients with hypoparathyroidism was lower than that of patients with normoparathyroidism.

## Figures and Tables

**Table 1:**
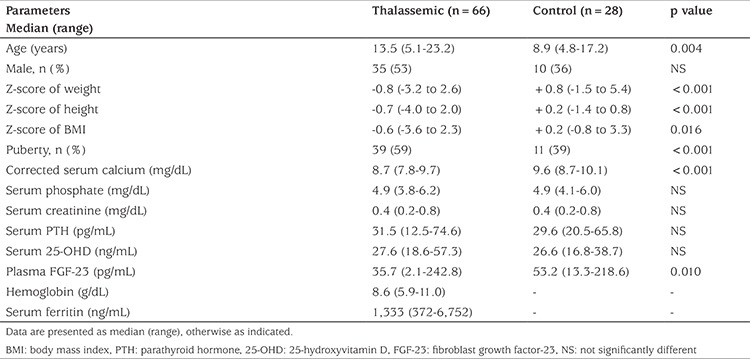
Characteristics and blood chemistry of thalassemic patients and controls

**Table 2:**
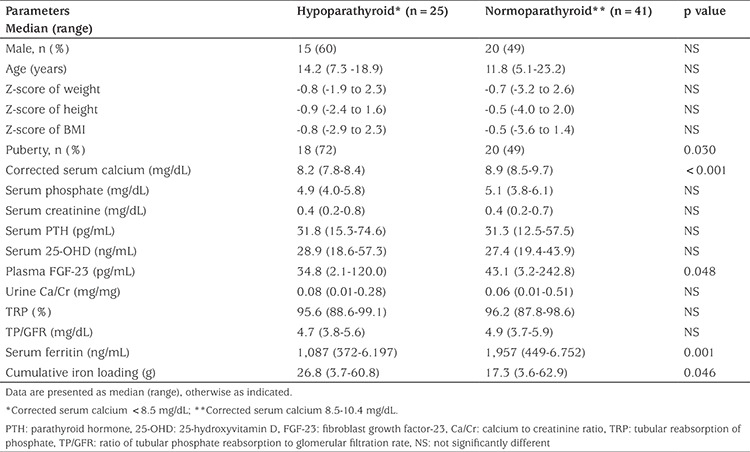
Anthropometric and biochemical parameters in hypoparathyroid and normoparathyroid thalassemic patients
